# A chemical language model for molecular taste prediction

**DOI:** 10.1038/s41538-025-00474-z

**Published:** 2025-07-05

**Authors:** Yoel Zimmermann, Leif Sieben, Henrik Seng, Philipp Pestlin, Franz Görlich

**Affiliations:** 1 Department of Chemistry and Applied Biosciences, ETH Zurich, Zurich, Switzerland; 2 Kvant AI Labs, Zurich, Switzerland

**Keywords:** Chemistry, Nutrition

## Abstract

Determining molecular taste remains a significant challenge in food science. Here, we present FART (Flavor Analysis and Recognition Transformer), a chemical language model capable of predicting molecular taste from chemical structure. Trained on the largest public dataset (15,025 compounds) of molecular tastants to date, FART is the first model capable of parallel predictions across four taste categories: sweet, bitter, sour, and umami. FART achieves an accuracy above 91% for parallel taste prediction and outperforms previous state-of-the-art binary classifier models that specialize on predicting one taste class. Its transformer architecture allows for interpretability through gradient-based visualization of molecular features. The model identifies key structural elements driving taste properties and demonstrates utility in analyzing known tastants as well as novel compounds. By releasing both the model and dataset, we equip the food science community with tools for rapid taste prediction, accelerating flavor compound development and enabling systematic exploration of taste chemistry.

## Introduction

Flavor sensation is a complex phenomenon in which concentration, prior perceptions, cultural and immediate context, individual physiology, the combination of various tastants, as well as other sensations such as smell or texture play an important role^1–3^. Determining the taste of even a single molecule in isolation remains a time- and labor-intensive process involving human panelists or electronic tongues^4,5^. Computational tools capable of predicting the taste of a molecule in silico could meaningfully accelerate this task and assist in the discovery of novel tastants.

A molecule elicits a taste perception by interacting with taste receptors in the mouth as determined by its steric and electronic properties^6^. As a result, the taste of a molecule should be a function of its underlying chemical structure. Over the past decade, machine learning methods have repeatedly demonstrated the ability to computationally predict molecular properties when provided with sufficient data^7^. By learning the relationship between molecular data and chemical structure, these models can predict properties even for compounds for which no data was initially available.

In recent decades, a number of machine learning approaches have been suggested for molecular taste prediction, as has been helpfully reviewed elsewhere^8–10^. Very recently, large language models such as OpenAI’s GPT-3.5 and GPT-4 have been used for taste prediction^11,12^, although it is unclear how well these proprietary models generalize to molecules not contained in their training data^13^. Fundamentally, the performance of machine learning methods is limited by the quality and size of the dataset available. Here we curated the largest, public dataset of 15,025 molecules and their associated taste labels, which is fully accessible in accordance with the FAIR principles^14^.

**Fig. 1:**
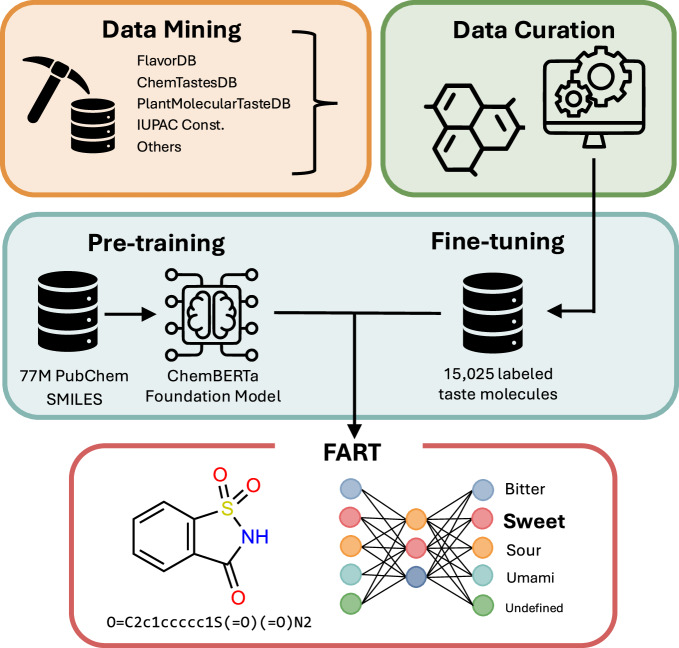
Schematic representation of the methodology behind the Flavor Analysis and Recognition Transformer (FART), showcasing the integration of data mining, data curation and fine-tuning.

Using this dataset, we developed a chemical language model using the transformer architecture^15^, named Flavor Analysis and Recognition Transformer (FART). This new model compares favorably to baseline machine learning methods in a multi-class setting. FART is the first chemical language model developed for molecular taste prediction. While FART is trained to predict four taste classes in parallel, it consistently outperforms models specifically designed for binary classification tasks, e.g. sweet/non-sweet or bitter/non-bitter. Central to the success of transformer models is the pre-training, fine-tuning paradigm^16,17^. In natural language processing (NLP) for which the transformer architecture was originally developed, a model is first pre-trained on a large corpus of text allowing it to learn general language structures and semantic relationships. This broad foundation is then refined through fine-tuning on smaller, domain-specific datasets, adapting the model to excel at specialized tasks. Analogously, FART is based on a pre-trained foundation model for chemistry, ChemBERTa^18,19^, which was then fine-tuned with the dataset presented here, see Fig. 1. Instead of natural language, a chemical language model takes SMILES (Simplified Molecular Input Line Entry System)^20^ as input, a text-based notation that encodes chemical structures into linear strings. FART is hence capable of predicting taste from chemical structure alone, obviating the need for further data collection from either experiments or computations.

Unlike previous approaches, which were limited to predicting one to two taste classes, FART is capable of classifying molecules across four of the basic human taste categories: sweet, bitter, umami and sour. The category of “salty" was excluded as salty taste is principally mediated by physical properties such as ion size and solubility rather than molecular structure^21^, precluding structure-based molecular taste prediction models. Instead, we opted to include an additional category “undefined", which combines all molecules which cannot be assigned to any of the four taste categories. The category of “undefined" hence encompasses salty, tasteless as well as molecules with ambiguous taste profiles.

FART is the first chemical language model for taste prediction of small molecules capable of predicting the four common taste categories in parallel. To be most useful for food scientists, such a machine learning model should not behave as a black box and allow for at least some rationalization of its predictions. The transformer architecture offers opportunities for interpretability through an analysis of its attention mechanism^15,22^ or gradient-based interpretability methods. The latter evaluates how changes in the input (such as atomic structure) impact the model’s predictions, attributing the result to specific features^23^. Using SMILES as input further allowed for the deployment of a confidence metric to quantify the uncertainty of the model’s predictions. In the future, robust and interpretable taste prediction could advance drug formulation and rational food design as well as help elucidate the interaction of molecules with their respective taste receptors.

## Results

The performance of the models was evaluated on a subset of 15% (or 2254 molecules) of the dataset, which had been excluded from the training. The test set used for evaluation constitutes an independent, representative, and randomized subsample of the full dataset. Because molecules were deduplicated, no molecules seen during training exist in the test set. All models were evaluated on the same test set for various metrics, see Table 1. While accuracy measures how a model performs across all five taste categories, other metrics need to be calculated on a per-class basis. For the class “sweet" for example we would evaluate *precision* (proportion of predicted sweet molecules which are truly sweet), *recall* (proportion of truly sweet molecules that were also predicted sweet), the *F1 score* (which combines recall and precision into one number) and the area under the receiver operating characteristic (AUROC, which measures a model’s ability to distinguish between classes). As FART is specifically tasked with parallel predictions across four taste categories, we decided to report overall performance using unweighted (macro) averages across the five taste categories. This choice means that a model cannot compensate bad performance on a minority class (in our case umami) with exceptionally good performance on the other, more common classes. More detailed results for each taste class individually and their weighted averages can be found in the supplementary information (see, Supplementary Tables 2–9).

**Table 1:** Performance comparison between the trained transformers and random-forest models across several metrics

		Unweighted average				
Model	Accuracy	Precision	Recall	F1 Score	AUROC	Support
XGBoost: fingerprints (fp)	0.8988	0.8169	0.7400	0.7661	0.8520	100%
XGBoost: fp+descriptors	0.8962	0.8842	0.7402	0.7779	0.8513	100%
Balanced Random Forest: fp	0.7972	0.5845	0.7322	0.6014	0.8391	100%
Chemprop	0.8851	0.8037	0.7224	0.7499	0.8392	100%
FART	0.8860	0.7720	0.6873	0.7118	0.9639	100%
FART augmented	0.8940	0.8789	0.7388	0.7737	0.9744	100%
FART augmented + confidence	**0.9155**	**0.9000**	**0.7617**	**0.7956**	**0.9806**	94%

All scores are given as unweighted averages across taste classes which emphasizes minority classes, in our case umami. Area under the receiver operating characteristic (AUROC) values are calculated as one-vs-rest for each taste class. Support refers to the number of compounds out of the test set which were assigned a prediction by the model. The best score is highlighted for each metric in bold.

We compared FART to tree-based classifier models (XGBoost, Random Forest) which are considered strong baselines for classification tasks compared to more complex model architectures such as transformers^24^. This benchmarking relative to simpler, tree-based classifiers, which often perform exceptionally well with tabular data, is necessary to assess whether the added complexity of the transformer architecture is justified. We also included another deep learning model, Chemprop^25^, which is a message-passing neural network (MPNN) using graphs as molecular representations. Chemprop uses no pre-training but has repeatedly shown strong performance in molecular property prediction tasks^7^.

### Baseline tree-based classifiers

Notably, the balanced random-forest method, which under-samples the majority class to ensure that all classes are equally represented during training, did not perform better even when considering the more favorable unweighted averages. XGBoost^26^ combined with a larger albeit unbalanced dataset yielded superior prediction performance. Unlike the transformer models, which are trained on text-based representations of molecules (i.e. SMILES), the baseline classifiers were trained using a vector representation of molecules known as Morgan fingerprints^27^. Concatenating these fingerprints with an additional set of 15 molecular descriptors that were previously found to be highly correlated with taste^28^ did not improve performance. This observation is relatively unsurprising given that these descriptors are nearly exclusively based on adjacency matrices and therefore encode little additional information compared to the atom-radius based Morgan fingerprint.

### Transformer models

Fine-tuning on the curated dataset led FART to a performance already on par with the best tree-based models showing that fine-tuned transformer models are capable of taste prediction given sufficient data, see Table 1. A common technique to synthetically expand the dataset to improve generalizability is to use SMILES augmentation. By using the fact that the same molecule can be represented by multiple SMILES strings, it is possible to generate such additional non-canonical SMILES for each data point^29^. SMILES augmentation can help improve performance more generally but is particularly useful in ensuring the model generalizes well to input SMILES which are not canonicalized. Indeed, the performance of the unaugmented iteration of FART dropped markedly (by about 6–10 percentage points across metrics) when non-canonical SMILES were used as input, whereas the performance of the augmented model remains essentially unchanged (see Supplementary Tables 10 and 11 as well as Supplementary Fig. 3 in the SI). Training FART on this augmented dataset resulted in improved performance and made predictions much more robust towards different SMILES of the same molecule. The biggest boost in performance was actually realized for the minority class of umami, where the F1-score increased from 0.25 to 0.50 upon using augmentation, see Supplementary Tables 6–9 in the SI. Furthermore, this augmentation allowed us to construct a confidence metric in which a prediction is only accepted when a consensus is reached across 10 different SMILES representations for the same molecule, see also Section “Confidence Metric”. Notably, data augmentation cannot be used to address data imbalance in the training set. We found that when augmenting in an imbalanced manner, i.e. mostly augmenting our minority class of umami and not augmenting our majority class of sweet, FART greedily classified all canonicalized SMILES as sweet. Performance as measured by a one-vs-rest AUROC consistently improved for FART models compared to XGBoost baselines, which suggests that FART learned an expressive latent representation of the input molecules in which taste classes can be more easily distinguished. The Receiver Operating Characteristics (ROCs) are shown in Supplementary Fig. 2 in the SI.

To further compare the performance of FART to other deep learning methods, we also trained a Chemprop model on the same dataset. Chemprop^25^ is a widely used model for chemical property prediction and is based on a directed message-passing neural network (D-MPNN) architecture that uses graph representations of molecules, rather than the text-based SMILES representation, as input. We find that the Chemprop model performs similarly to the unaugmented FART model as well as the XGBoost baseline. The FART model trained on the augmented dataset and using a confidence metric remains the best performing model. The fact that SMILES, unlike graphs, are easily augmented enabled the use of a confidence metric that meaningfully increases the reliability of predictions without adding major computational overhead.

Molecular tastants are not confined to a single label however, the archetypical example being bittersweet compounds^30^. In the FART dataset, such compounds led to repeated entries with the same canonicalized SMILES for each taste associated. Indeed, of the 409 molecules in the dataset with multiple tastes associated, 152 (37%) only have “undefined" as an additional label and 165 (40%) are labeled as bittersweet. Because FART outputs a probability distribution across the taste labels, one could expect the probability to be higher for the two (and in rare cases three) original labels compared to the other taste classes. In other words, a bittersweet molecule should have higher probabilities for both bitter and sweet while ignoring the remaining classes. We find that in most cases FART strongly favors one out of the two or more labels, which is unsurprising given the model was also trained and evaluated on single labeled data points.

Molecules that could be associated with multiple tastes during data curation are given as duplicates in the dataset with the same canonicalized SMILES but different taste labels. To test how FART augmented evaluates on these multi-label molecules, we considered all labels above a probability of 0.2, i.e. higher than a uniform distribution across the classes, as relevant rather than considering the highest probability as done for normal evaluation. Compounds which only had “undefined" as an additional label were excluded. Of the 257 remaining multi-class molecules, which were all seen during training, FART correctly identifies the correct labels exactly for just 25 molecules. In 20 cases, FART associates too many labels. In all of these cases the additional label is “undefined". Overwhelmingly, however, FART collapses the multiple labels into a single one which happens for 121 molecules. In 108 of these cases, only a single but correct label was predicted, see also Supplementary Fig. 5 in the SI. Ultimately, during training as well as inference, FART is tasked with producing a single output label which helps explain why the models struggle with this parallel multi-class prediction task. More work is needed to develop models that more accurately reflect the nature of multi-class tastants.

The advantage of including an “undefined" class for taste prediction is that FART is capable of flagging tasteless molecules as well as compounds that are plausibly outside the domain of application, i.e. molecular structures that are too distinct from the train set to allow for meaningful predictions. For example, instead of forcing sodium chloride erroneously into one of the four taste labels that we have collected data on, FART classifies it as undefined. Similarly, FART classifies saturated alkanes, which are not water-soluble, as undefined as well.

We find that the best FART model trained on the augmented dataset and using a confidence metric results in an accuracy above 90%, outperforming both tree-based baseline models such as XGBoost as well as other deep learning methods such as Chemprop. The improved performance of FART justifies the added computational complexity relative to baseline models. FART remains efficient in both training and inference by relying on tokenization instead of computationally expensive graph construction, employing a lightweight confidence metric based on SMILES augmentation, and utilizing a comparatively fast underlying language model. Nonetheless, we were curious how FART, which predicts five taste labels in parallel, compares to previous models that were specifically trained on a single taste label.

### Comparison to binary classifiers

Compared to state-of-the-art classifiers tasked with binary taste prediction (sweet/non-sweet, bitter/non-bitter, sour/non-sour or umami/non-umami), FART consistently performs better despite having been trained on the more challenging task of multi-class prediction, see Table 2. Note that while the performance metrics were re-calculated for FART based on the respective binary prediction tasks, the test set FART was evaluated on is significantly larger (2254 vs below 500) and more diverse than the homogeneous test sets compiled for binary prediction such as sweet/non-sweet. Therefore, FART has to learn a much broader chemical space and has to embed information for five rather than two labels. Despite this, FART performs superior compared to these state-of-the-art binary classifiers.

**Table 2:** Comparison of the FART models with previously published work using state-of-the-art binary classifiers as given in refs. ^9,46,55–59^

	Reference	Model name	Classifier	*n*	Accuracy	F1	AUROC
Sweet/non-sweet	Tuwani et al.^55^	BitterSweet	AdaBoost	161	0.834	0.856	0.883
	Fritz et al.^56^	VirtualSweet	RF	403	0.893	0.888	0.951
	Bo et al.^57^	SweetMLP-Fingerprint	MLP	444	0.900	–	0.940
	Lee et al.^58^	BoostSweet	Consensus	459	0.899	0.907	0.961
	Yang et al.^59^	ChemSweet	RF	241	0.920	–	0.971
	This work	FART augmented	Transformer	2254	0.926	0.944	0.978
	This work	FART confidence	Transformer	2129	**0.938**	**0.954**	**0.984**
Bitter/non-bitter	Tuwani et al.^55^	BitterSweet	RF	154	0.819	0.838	0.880
	Fritz et al.^56^	VirtualBitter	RF	323	0.898	**0.882**	0.956
	Charoenkwan et al.^46^	BERT4Bitter	Transformer	128	0.922	–	0.964
	Bo et al.^57^	BitterMLP-Descriptor	MLP	446	0.820	–	0.940
	This work	FART augmented	Transformer	2254	0.958	0.778	0.951
	This work	FART confidence	Transformer	2129	**0.970**	0.830	**0.965**
Sour/non-sour	Fritz et al.^56^	VirtualSour	RF	133	0.977	0.842	0.994
	This work	FART augmented	Transformer	2254	0.980	0.906	0.994
	This work	FART confidence	Transformer	2129	**0.986**	**0.935**	**0.997**
Umami/non-umami	This work	FART augmented	Transformer	2254	0.998	0.5	0.989
	This work	FART confidence	Transformer	2129	**0.998**	**0.5**	**0.989**

The best scores in each taste category are highlighted in bold. Despite being trained for multi-class prediction, FART outperforms state-of-the-art methods specifically trained on predicting only sweet/non-sweet, bitter/non-bitter, sour/non-sour and umami/non-umami, respectively. The size of the test set (*n*) is also reported.

*RF* Random forest, *MLP* Multi-layer perceptron.

### Interpretability

To better understand how FART arrives at its predictions, we implemented an interpretability technique based on integrating the gradients of the neural network’s output along the path from a baseline input (the zero embedding) to the input at hand^23^. For FART, this enables the visualization of atoms and functional groups that contribute positively toward the prediction of a given label (e.g., “sour") in green, while those that detract from this prediction are highlighted in red. We found that in many cases this analysis reproduces previously known patterns in food chemistry.

**Fig. 2:**
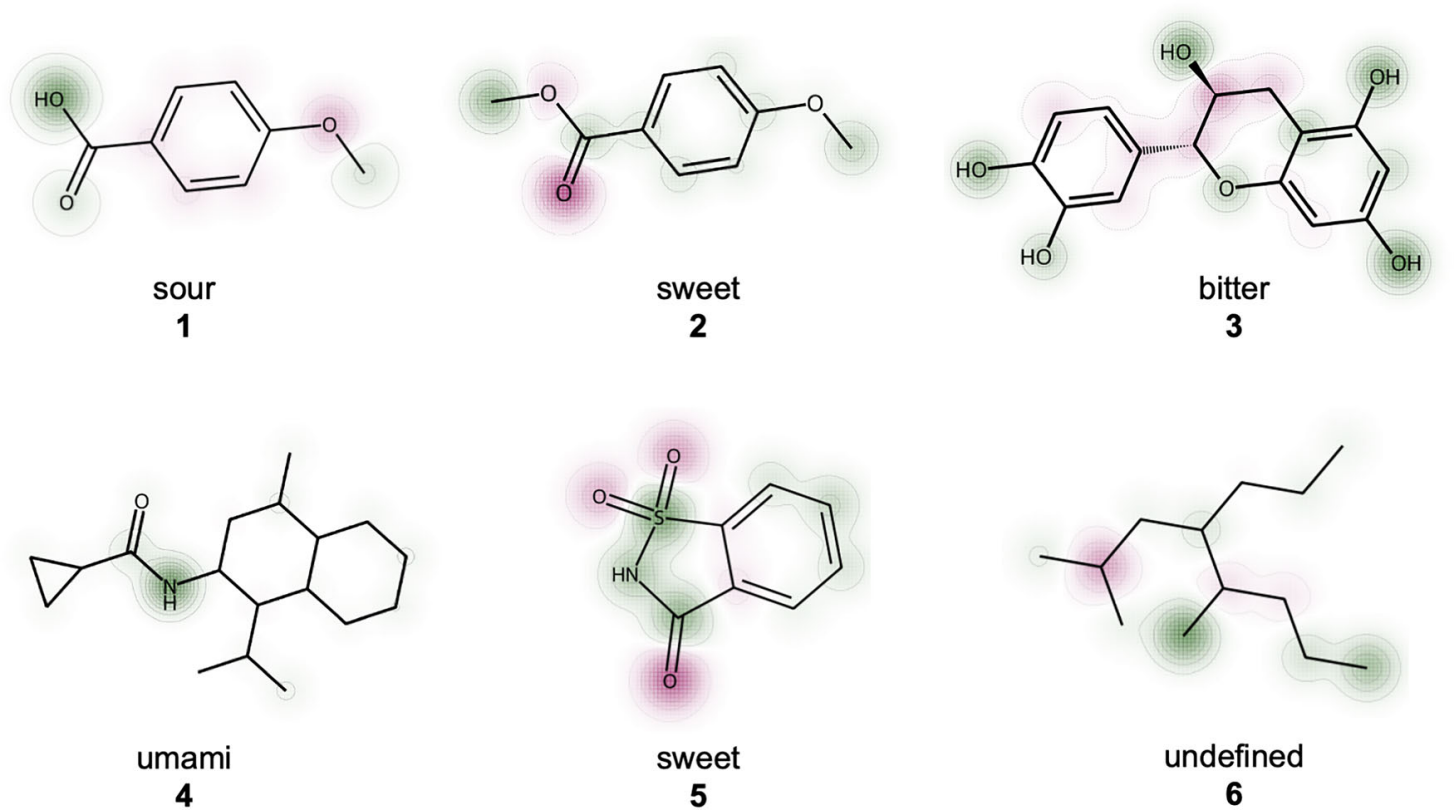
Using the integrated gradients method, atoms can be highlighted according to how important they were for classification. Positive contributions regarding the assigned label are highlighted in green, and negative ones in red. *p*-Anisic acid (**1**) is correctly classified as sour (pK_*a*_ 4.47) and yields methyl anisate (**2**) after esterification, which is found in star anise and has a sweet and fruity flavor. Catechin (**3**) is a bitter member of the polyphenol subgroup called flavenoids. The amide (**4**) is an analog of an umami flavor enhancer called cyclopropanecarboxylic acid (2-isopropyl-5-methyl-cyclohexyl)-amide. Saccharin (**5**) is a common artificial sweetener and 2,5-dimethyl-4-propyloctane (**6**) is a water unsoluble alkane that is not known to elicit any taste.

To test this interpretability framework, we deployed FART on six compounds with known labels as shown in Fig. 2, where two (compounds **4** and **6**) were not in the dataset collected in this work. For molecules labeled as sour for example, FART typically highlights the acid group, as is the case for *p*-anisic acid (**1**). Notably, even upon a small chemical change of essentially one carbon atom, where the acid group is esterified to yield methyl anisate (**2**), FART reassigns a new label which can also be seen in the integrated gradients for the new compound. The methyl group of the ester is now relevant for the predicted sweet taste. This assignment has some grounding in truth as esters broadly, and methyl anisate in particular, are common flavor and odor chemicals with typically fruity flavors and aromas, which would likely be labeled as sweet by a human panelist^31,32^.

Similarly, FART gave higher weight to the polyphenol or more specifically the flavenoid scaffold of catechin (**3**) which is a shared substructure among many bitter-tasting compounds^33^. This approach to interpretability does also extend to molecules that were not part of our dataset, such as compound **4**, which is an analog of a known umami tastant, cyclopropanecarboxylic acid (2-isopropyl-5-methyl-cyclohexyl)-amide but which does not exist in the dataset. Interestingly, the amide group, a feature of many umami molecules, particularly peptides, is overwhelmingly responsible for this classification. While sensible in the context of this molecule, this prediction also highlights some of the limitations of this approach as an amide group alone is not sufficient to elicit an umami taste. For the artificial sweetener saccharine (**5**), the sultam functional group with its sulfur-nitrogen bond is highlighted which is a pattern shared for example with another sweetener, cyclamate^34^. Seemingly, the model negatively attributes oxygen groups for this label, possibly because these oxygen double bonds are more associated with the label “sour", as this motif prominently features in many acids such as carboxylic or sulfuric acid. This might similarly explain the same negative attribution for methyl anisate (**2**).

Even in well-behaved cases, however, not every contribution in the resulting heatmap may be individually interpretable or chemically intuitive. In the case of saccharine for example, it is less clear why the oxygen atoms are particularly anti-correlated with sweetness. Similarly for the alkane **6**, which also does not feature in the dataset, it is rather the absence of any features that leads to it being classified as undefined. Nonetheless, these interpretability tools help rationalize FART’s predictions and may aid in producing analogs by highlighting parts of the structure that can be altered without changing the label, see Supplementary Fig. 4 in the SI for an example use case. While FART achieves very high overall accuracy, certain general properties of tastants are not well modeled. Furthermore, despite stereochemistry being encoded in SMILES, FART generally lacks sensitivity to stereocenter inversions. For example, while the natural stereoisomer L-glutamate has an umami taste, its counterpart D-glutamate is tasteless^35^. FART incorrectly categorizes both compounds as having umami flavor.

## Discussion

The chemical language model FART is able to compete with state-of-the-art taste prediction models and baseline tree-based methods. Enabled by the fine-tuning on a large training set of 15,025 labeled small molecule tastants, FART is the first model capable of predictions across all four major taste categories (sweet, sour, bitter, umami) while summarizing other molecules into a fifth category ("undefined"). FART provides robust, interpretable and fast predictions based on chemical structure alone, which avoids the computational overhead of calculating physical or chemical descriptors.

To the best of our knowledge, the dataset presented in this work includes the large majority of high-quality labeled taste data publicly available today and may serve as a useful resource for future work on small molecule-taste prediction. Nonetheless, the scarcity of data on umami compounds makes this a particularly challenging taste category also for FART and future work should focus on improving predictions for these rarer taste categories as well as extending these predictions to peptides and other biological macromolecules. Future efforts should also focus on evaluating these models prospectively and experimentally validating taste predictions across different populations. Progress is already underway to build increasingly powerful chemical language models that could be fine-tuned using a similar approach as outlined here. However, we believe meaningful breakthroughs in taste prediction will not come from methodological advancements alone but require the collection of larger, well-curated, and accessible datasets. Automated, experimental taste classification remains a large unmet need in the field of food chemistry today and would enable the large-scale collection of data as well as rapid validation of model predictions.

An advantage of using language models pre-trained on chemical data is that they typically generalize better^18,36^ to unseen molecules which is particularly relevant when applying these models for the discovery of novel small molecule tastants. In the future, FART and methods like it may be used to perform in-silico screenings of large chemical spaces to discover novel tastants with desirable properties similar to modern drug discovery approaches^37^. Future work should also focus on improving model architectures so that molecules with multiple associated tastes are correctly assigned multiple labels as well as making FART more sensitive toward stereochemistry. We appreciate that the use of synthetic chemicals to enhance or alter food flavor is not universally desirable. However, machine learning tools such as FART may also plausibly aid food scientists during the analysis of natural foodstuffs by shortlisting flavor-rich small molecules from a mixture of compounds. In this way, only a small number of promising compounds would need to be experimentally validated. Such an approach is highly dependent on the robustness of prediction and would motivate extending these predictions to peptides and other macromolecules as well.

Artificial intelligence has a role in assisting the food scientist of tomorrow. Taste prediction tools offer great utility for the discovery, development as well as analysis of small molecule tastants even if they cannot replace the need for thorough experimental validation. Machine learning tools like FART could open up new and exciting chemical spaces for food scientists going forward, helping bring molecular food science into the age of big data and deep learning.

## Methods

### Dataset

One aim of this work was to curate a large, high-quality dataset of molecular tastants from publicly available data. Every molecule was assigned a taste label: sweet, bitter, sour, umami, or undefined. The category of “undefined" contains molecules, not clearly assignable to one of the other categories, explicitly including compounds labeled as salty or tasteless. The dataset utilizes a standard text-based representation of molecules, called SMILES^20^. Every molecule is labeled with a taste: sweet, bitter, sour, umami, and undefined. Where the last category encompasses compounds that had either previously been established as tasteless or compounds that were present in the source databases but for which no clear taste could be associated, particularly compounds with odor rather than taste labels. Salty was excluded as a taste category because only a very small number of molecules actually produce this taste apart from sodium and chloride ions^21^. Data curation was handled with the cheminformatics package RDKit^38^.

**Fig. 3:**
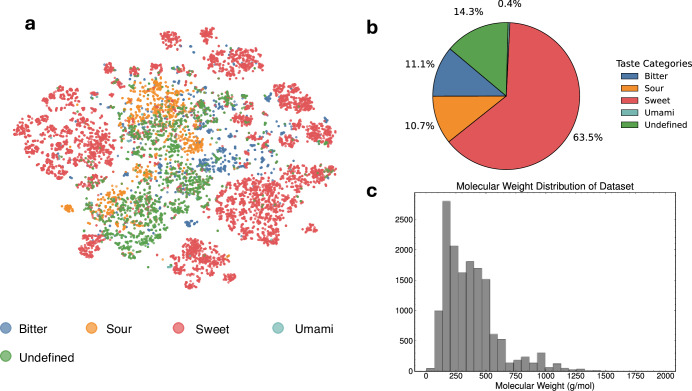
**a** t-SNE plot of the chemical space covered by the dataset. **b** Distribution across taste classes for the dataset, showing a strong imbalance for umami. **c** Molecular weight distribution for the dataset (mean 374 g/mol, std. deviation 228 g/mol).

The FART dataset encompasses small molecules with an average molecular weight of 374 Da ±228, see Fig. 3c. To visualize the distribution of the taste labels over the chemical space covered by the dataset, a t-SNE plot (Fig. 3a) was generated from Morgan fingerprints^27^. Figure 3b highlights the strong imbalance of the taste classes of the dataset.

The FART dataset aggregates experimental data from six sources^39–43^, see also Supplementary Table 1 in the SI. ChemTasteDB is one of the largest public databases of tastants and contains 2944 organic and inorganic tastants from which 2177 were used to train FART. The database was curated from literature^39^. Biological macromolecules were generally excluded, notably longer peptides, which we do not consider in our approach. Previous work has explored this chemical space particularly in the context of umami and bitter prediction^44–46^.

FlavorDB aggregates data on both gustatory and olfactory sensation from a number of sources^40^. The FART database uses the “flavor profile" given by FlavorDB as most molecules do not have a specific entry for taste. Data from FlavorDB will thus be more heterogeneous given that some of these flavor profiles will actually be based on smell, not taste. Care was taken to only include compounds with unambiguous taste adjectives in the dataset. From the 25,595 total molecules, 10,372 could be clearly attributed to one of the four taste categories. FlavorDB is dominated by sweet molecules and is also the source of the data imbalance in the final dataset. PlantMolecularTasteDB contains 1,527 phytochemicals with associated taste of which 906 were used for this dataset^41^. The database is based on both literature and other databases, some of which overlap with other sources used for FART. To obtain more data on bitter compounds, a database of ligands that bind to the human bitter receptor (TAS2) was also considered which yielded 53 previously unseen bitter compounds^42^. Combined, these datasets represent the largest publicly available dataset of unique, labeled molecule-taste pairs. However, there remains significant heterogeneity in how these datasets reference results, how experimental data was collected, and certain biases in terms of cultural or genetic preferences may persist. Thorough curation is thus needed to guarantee standardized data and to reduce noise as much as possible.

Water-soluble, acidic molecules (pK_A_ between 2 and 7), assumed to taste sour^47^, were collected from an ongoing project based with the International Union of Pure and Applied Chemistry (IUPAC) digitizing three high-quality sources of pK_A_ values in the literature^48–50^. Sour taste is influenced by other factors such as cell permeability, which is the reason why organic acids taste more acidic than inorganic acids such as HCl at the same pH. Nonetheless, acidic molecules can be assumed to also taste sour^47^. A total of 1,513 acids could be obtained in this way although it should be noted that sour taste, as all tastes, is concentration-dependent and that some of the weaker acids may not be picked up by humans. The pK_A_ values refer to the most acidic proton and are all measured between 15 and 30 ^∘^C in water, i.e. around physiological temperature, excluding any acids that are not water-soluble. Lastly, 19 umami-tasting molecules were collected from the literature^43^ of which 11 were not found in any other database.

The combined dataset was reduced to the taste label associated with a canonicalized SMILES representation. The open-source cheminformatics package RDKit^38^ was used to further curate the dataset. First, all SMILES that did not allow the generation of a valid molecular graph were excluded. To avoid solvent-containing molecules, all entries with multiple uncharged fragments were removed. Charged molecules were additionally excluded to prevent substances with missing counter ions. Labels were visually inspected and manually corrected for around 100 molecules. All SMILES were standardized with the default RDKit standardization procedure including canonicalization. Duplicates could be removed with the help of these canonicalized SMILES, see Supplementary Fig. 1 in the SI for a summary.

While only very few entries with invalid SMILES (21) or charged molecules (342) needed to be removed, the number of entries containing multiple neutral fragments (3783) was more significant. These are typically SMILES that contain the tastant as well as a solvent which needs to be removed. Filtering for molecules below 2000 Dalton in molecular weight (1) was introduced to avoid SMILES strings that are longer than what the context window of ChemBERTa allows. The dataset was further inspected by eye and some mislabeled entries were manually corrected. The duplicate removal (14,685) reduced the dataset by almost half to a final size of 15,025 entries, see Supplementary Fig. 1. The large number of duplicates underlines the significant overlap among the databases used. When duplicate entries existed from different sources, which source would be given in the final dataset was arbitrarily determined based on the index. The final dataset exhibits a strong data imbalance, where sweet represents over 60% and umami less than 1% of the data.

The curated dataset was further enriched by general information (PubChemID, IUPAC name, molecular formula, molecular weight, InChI, InChIKey), accessed through the PubChem API^51^. The dataset, FartDB, was published in agreement with the FAIR principles^14^ and can be accessed through several different interfaces to encourage its use by other research projects.

### Visualization

The t-SNE plot, see Fig. 3a, (perplexity = 30) was generated using 1024-Morgan fingerprints (radius = 2) based on PCA initialization and the Jaccard distance metric. Heatmap plots for interpretability were generated using custom code utilizing the SimilarityMaps functionality of RDKit, see Fig. 2.

### Tree-based classifiers: XGBoost, random forest

Tree-based ensemble models such as random forest (RF) or XGBoost^26^ are often considered strong baseline models in classification tasks for more complex model architectures, such as transformers, given their robust performance, efficient training, and low model complexity. A RF model is an ensemble learning method that trains multiple, in this case 150, decision trees during the training process, combining their predictions to improve accuracy and mitigate overfitting on the training data. A XGBoost model is an ensemble learning method that builds multiple decision trees sequentially, optimizing each tree to correct errors from the previous ones, thereby improving accuracy and mitigating overfitting through regularization techniques.

In this work, three different tree-based classifiers were trained including hyperparameter optimization. The two XGBoost models were trained either using 1024-Morgan (radius = 2) fingerprints or these same fingerprints concatenated with 15 descriptors calculated using Mordred^52^. These predictors had been previously found to be particularly correlated with taste^28^. The models were evaluated with a multi-class logarithmic loss function, results are given in Table 1.

### FART models

For our transformer-based model, we utilized ChemBERTa^18,19^, which has been pre-trained on 77 million SMILES strings using a masked language modeling approach. Here, a percentage of the input is randomly masked, and the model is trained to predict the masked parts of the SMILES string. This allows the model to learn contextualized representations of chemical structures in a self-supervised manner, capturing both local and global molecular features. We fine-tuned ChemBERTa on our taste prediction dataset using a categorical cross-entropy loss function with a learning rate of 10^−5^ and a batch size of 16. We experimented with three different training configurations: one model was trained for 20 epochs on the original dataset and a second model was trained for 2 epochs on a 10-fold augmented dataset. The performance of all models is summarized in Table 1. The Chemprop model were trained as described in the original publication using default hyperparameter optimization^25^.

To evaluate multi-class classification performance, macro and weighted averages are commonly used to summarize metrics across all classes. The macro (unweighted) average is computed with

1 $${\rm{Macro}}=\frac{1}{C}\mathop{\sum }\limits_{i=1}^{C}{M}_{i},$$

where *C* is the number of classes and *M*_*i*_ is the metric (e.g., precision, recall, F1-score) for the *i*th class. The weighted average is given by

2 $${\rm{Weighted}}=\mathop{\sum }\limits_{i=1}^{C}\frac{{n}_{i}}{N}\cdot {M}_{i},$$

where *n*_*i*_ is the number of instances in class *i*, *N* is the total number of instances across all classes, and *M*_*i*_ is the metric for the *i*th class.

All transformer models were trained on multiple NVIDIA T4 GPUs in Google Cloud using the HuggingFace Transformers library^53^. For all experiments, the ChemBERTa checkpoint seyonec/SMILES_tokenized_PubChem_shard00_160k on HuggingFace was used, consisting of 6 layers and a total of 83.5 million parameters. Training on the unaugmented dataset was run for 20 epochs, while training on the augmented dataset was run for 2 epochs. A weight decay of 0.01 was applied, and a batch size of 16 was used. Both parameters follow standard values for fine-tuning and have not been optimized for this problem. Standard values for fine-tuning were also used for all other necessary parameters. Training was continued until overfitting was observed, as indicated by the loss function on the evaluation dataset, or until the loss had saturated. At this point, the best model checkpoint, corresponding to the lowest evaluation loss, was selected for further analysis.

### Confidence metric

Similar to linguistic synonymy, where multiple words share the same meaning, distinct SMILES representations can map to the same underlying molecular structure. To leverage this property, we generated an ensemble of 10 synonymous SMILES for each molecule in our dataset. The exact number of SMILES augmentation has been arbitrarily set and could alternatively be treated as an optimizable hyperparameter. The exact number of augmentations is arbitrary and can be defined by the user while ten is a reasonable number to expect even for smaller molecules. We then performed inference on the entire ensemble, obtaining individual predictions for each SMILES variant.

To aggregate these results, we employed a voting procedure across the ensemble’s predictions. Using the strictest threshold, where all 10 predictions had to agree for a label to be assigned by the model, we still retained predictions for 94% of the dataset while boosting the model’s accuracy to above 91%. Both the transient augmentation and subsequent prediction on these SMILES adds to the computational time. The confidence metric suggested here is straightforward to implement and provides a robust indication of prediction reliability in addition to the interpretability framework.

### Interpretability framework

Integrated gradients^23^ is a method for attributing a deep neural network’s prediction to its input features. The core idea is to integrate the gradients of the output taken along a linear path from a baseline input to the input at hand, see Eq. (3). Mathematically, for a neural network *F*(*x*), an input *x* and baseline input ${x}^{{\prime} }$ (e.g. the zero input), the attribution for the *i*th feature is:

3 $${{\rm{IntegratedGrads}}}_{i}(x)=\left({x}_{i}-{x}_{i}^{{\prime} }\right)\times \mathop{\int}\nolimits_{0}^{1}\frac{\partial F\left({x}^{{\prime} }+\alpha \times \left(x-{x}^{{\prime} }\right)\right)}{\partial {x}_{i}}\,{\rm{d}}\alpha .$$

The method satisfies important axioms like sensitivity (if inputs differ in one feature but have different predictions, that feature should receive attribution) and implementation invariance (attributions are identical for functionally equivalent networks). The method is readily available for Hugging Face Transformer models through the transformers-interpret package^54^.

## Supplementary information

Supplementary Information

## Data Availability

The data used and generated in our study is available at https://github.com/fart-lab/fart.git. All code used in this study is available at https://github.com/fart-lab/fart.git.
