# Supplementary material for: A chemical language model for molecular taste prediction
Source: NPJ Sci Food. 2025 Jul 5;9:122. doi: 10.1038/s41538-025-00474-z (PMC12228699; doi:10.1038/s41538-025-00474-z)
Supplement: Supplementary file 1 — Supplementary Information [file 41538_2025_474_MOESM1_ESM.pdf]

Supplementary Information for A Chemical Language Model for Molecular Taste Prediction

Yoel Zimmermann[†], Leif Sieben[†], Henrik Seng[†], Philipp Pestlin[†], and Franz Görlich[†]

Department of Chemistry and Applied Biosciences, ETH Zürich, Zürich, Switzerland

Kvant AI Labs, Zürich, Switzerland

[†]Equal Contribution

Contents

Supplementary Figures 1: Dataset Curation 2

Supplementary Table 2: Performance Overview as Weighted Averages 3

Supplementary Tables 3–5: Class-Resolved Performance Data for XGBoost and Random Forest 4

Supplementary Tables 6–9: Class-Resolved Performance Data for the FART and Chemprop models 5

Supplementary Figure 2: Receiver Operating Characteristics (ROC) for FART Models 7

Supplementary Tables 10-11: Comparison between augmented and unaugmented FART Models on Non-Canonical SMILES 8

Supplementary Figure 3: Receiver Operating Characteristics for Evaluation on Non-Canonical SMILES 9

Supplementary Figure 4: Analog Generation Based on Explainability Framework 10

Supplementary Figure 4: Analog Generation Based on Explainability Framework 11

Supplementary Figure 1 and Table 1: Dataset Curation

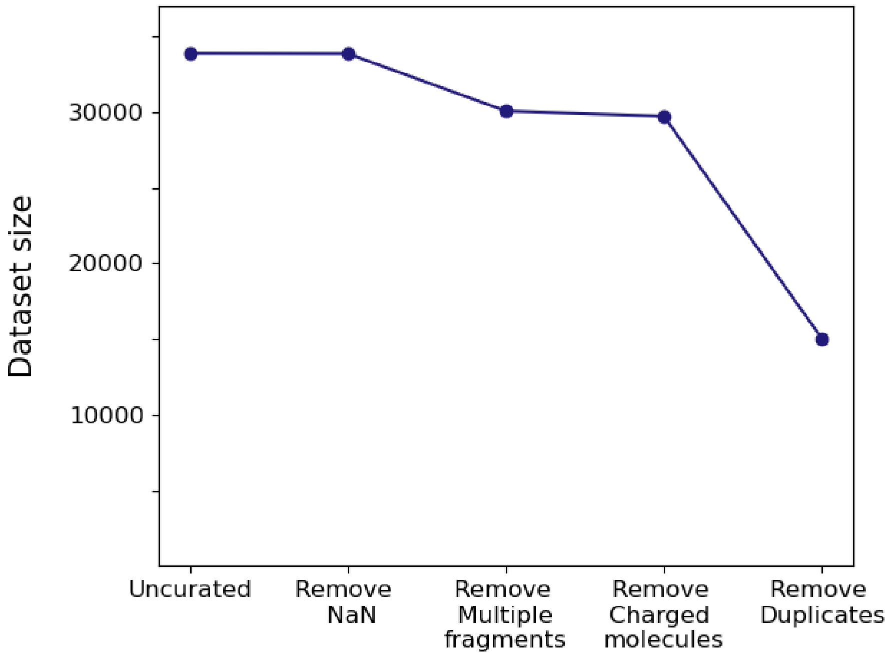

Supplementary Figure 1: The dataset size decreases during data curation. Duplicate removal nearly halved the size of the dataset.

Supplementary Table 1: Overview of the data sources used for FART.

Database	Sweet	Bitter	Sour	Umami	Undefined	Total
ChemTastesDB	832	956	19	50	338	2195
FlavorDB	8665	71	35	0	1601	10372
PlantMolecularTasteDB	87	615	40	1	138	881
TAS2R Agonists	0	53	0	0	0	53
IUPAC Dissociation Constants	0	0	1513	0	0	1513
Suess et al. 2015	0	0	0	11	0	11
Total	9584	1695	1607	62	2077	15025

Supplementary Table 2: Performance Overview as Weighted Averages

Supplementary Table 2: Performance comparison between the trained transformers and baseline classifiers. Scores are given as weighted averages across taste classes which penalizes wrong but rare predictions on a minority class less compared to an unweighted average. Scores for Random Forest and XGBoost were obtained through five-fold cross-validation. Area under the receiver operating characteristic (AUROC) values are calculated as one-vs-rest for each taste class and then combined into a weighted average.

		Weighted average				
Model	Accuracy	Precision	Recall	F1 Score	AUROC	Support
XGBoost: fingerprints (fp)	0.8988	0.8991	0.8988	0.8981	0.9098	100%
XGBoost: fp+descriptors	0.8962	0.8977	0.8962	0.8959	0.9059	100%
Balanced Random Forest: fp	0.7972	0.8393	0.7972	0.8079	0.8650	100%
Chemprop	0.8851	0.8817	0.8851	0.8820	0.8897	100%
FART	0.8860	0.8849	0.8860	0.8845	0.9737	100%
FART augmented	0.8940	0.8789	0.7388	0.7737	0.9744	100%
FART augmented + confidence	**0.9155**	**0.9192**	**0.9154**	**0.9159**	**0.9816**	94%

Supplementary Tables 3–5: Class-Resolved Performance Data for XGBoost and Random Forest

Supplementary Table 3: Class-resolved performance data for the XGBoost model trained on Morgan fingerprints with Fair Grid Search.

Taste Class	Accuracy	Precision	Recall	F1 Score	AUROC	Support
Bitter		0.8528	0.7210	0.7814		233
Sour		0.8893	0.9118	0.9004		238
Sweet		0.9434	0.9511	0.9473		1473
Umami		0.6667	0.3333	0.4444		6
Undefined		0.7323	0.7829	0.7568		304
Weighted Average		0.8991	0.8988	0.8981	0.9098	
Unweighted Average		0.8169	0.7400	0.7661	0.8520	
Overall	0.8988					2254

Supplementary Table 4: Class-resolved performance data for the XGBoost model trained on Morgan fingerprints and 15 additional descriptors.

Taste Class	Accuracy	Precision	Recall	F1 Score	AUROC	Support
Bitter		0.8571	0.7468	0.7982		233
Sour		0.9064	0.8950	0.9006		238
Sweet		0.9394	0.9464	0.9428		1473
Umami		1.0000	0.3333	0.5000		6
Undefined		0.7182	0.7796	0.7476		304
Weighted Average		0.8977	0.8962	0.8959	0.9059	
Unweighted Average		0.8842	0.7402	0.7779	0.8513	
Overall	0.8962					2254

Supplementary Table 5: Class-resolved performance data for the Balanced Random Forest model trained on Morgan fingerprints with future behavior.

Taste Class	Accuracy	Precision	Recall	F1 Score	AUROC	Support
Bitter		0.6852	0.4764	0.5620		233
Sour		0.6145	0.8908	0.7273		238
Sweet		0.9579	0.8344	0.8919		1473
Umami		0.0909	0.6667	0.1600		6
Undefined		0.5738	0.7928	0.6657		304
Weighted Average		0.8393	0.7972	0.8079	0.8650	
Unweighted Average		0.5845	0.7322	0.6014	0.8391	
Overall	0.7972					2254

Supplementary Tables 6–9: Class-Resolved Performance Data for the FART models

Supplementary Table 6: Class-resolved performance data for the Chemprop model on the unaugmented dataset.

Taste Class	Accuracy	Precision	Recall	F1 Score	AUROC	Support
Bitter		0.8283	0.7039	0.7610		233
Sour		0.8539	0.9580	0.9030		238
Sweet		0.9234	0.9491	0.9361		1473
Umami		0.6667	0.3333	0.4444		6
Undefined		0.7463	0.6678	0.7049		304
Weighted Average		0.8817	0.8851	0.8820	0.8897	
Unweighted Average		0.8037	0.7224	0.7499	0.8392	
Overall	0.8851					2254

Supplementary Table 7: Class-resolved performance data for the FART model trained on the unaugmented dataset.

Taste Class	Accuracy	Precision	Recall	F1 Score	AUROC	Support
Bitter		0.8030	0.6824	0.7378		233
Sour		0.9221	0.8950	0.9083		238
Sweet		0.9308	0.9491	0.9398		1473
Umami		0.5000	0.1667	0.2500		6
Undefined		0.7040	0.7434	0.7232		304
Weighted Average		0.8849	0.8860	0.8845	0.9737	
Unweighted Average		0.7720	0.6873	0.7118	0.9639	
Overall	0.8860					2254

Supplementary Table 8: Class-resolved performance data for the FART model trained on the augmented dataset.

Taste Class	Accuracy	Precision	Recall	F1 Score	AUROC	Support
Bitter		0.8557	0.7124	0.7775		233
Sour		0.8971	0.9160	0.9064		238
Sweet		0.9443	0.9430	0.9436		1473
Umami		1.0000	0.3333	0.5000		6
Undefined		0.6977	0.7895	0.7407		304
Weighted Average		0.8970	0.8940	0.8940	0.9750	
Unweighted Average		0.8790	0.7388	0.7736	0.9744	
Overall	0.8940					2254

Supplementary Table 9: Class-resolved performance under a confidence metric. Predictions are made only when the predicted label for 10 augmented SMILES agree.

Taste Class	Accuracy	Precision	Recall	F1 Score	AUROC	Support
Bitter		0.9059	0.7662	0.8302		201 (86%)
Sour		0.9292	0.9417	0.9354		223 (94%)
Sweet		0.9562	0.9522	0.9542		1445 (99%)
Umami		1.0000	0.3333	0.5000		6 (100%)
Undefined		0.7089	0.8150	0.7582		254 (84%)
Weighted Average		0.9192	0.9154	0.9159	0.9816	
Unweighted Average		0.9000	0.7617	0.7956	0.9806	
Overall	0.9155					2129 (94%)

Supplementary Figure 2: Receiver Operating Characteristics (ROC) for FART Models

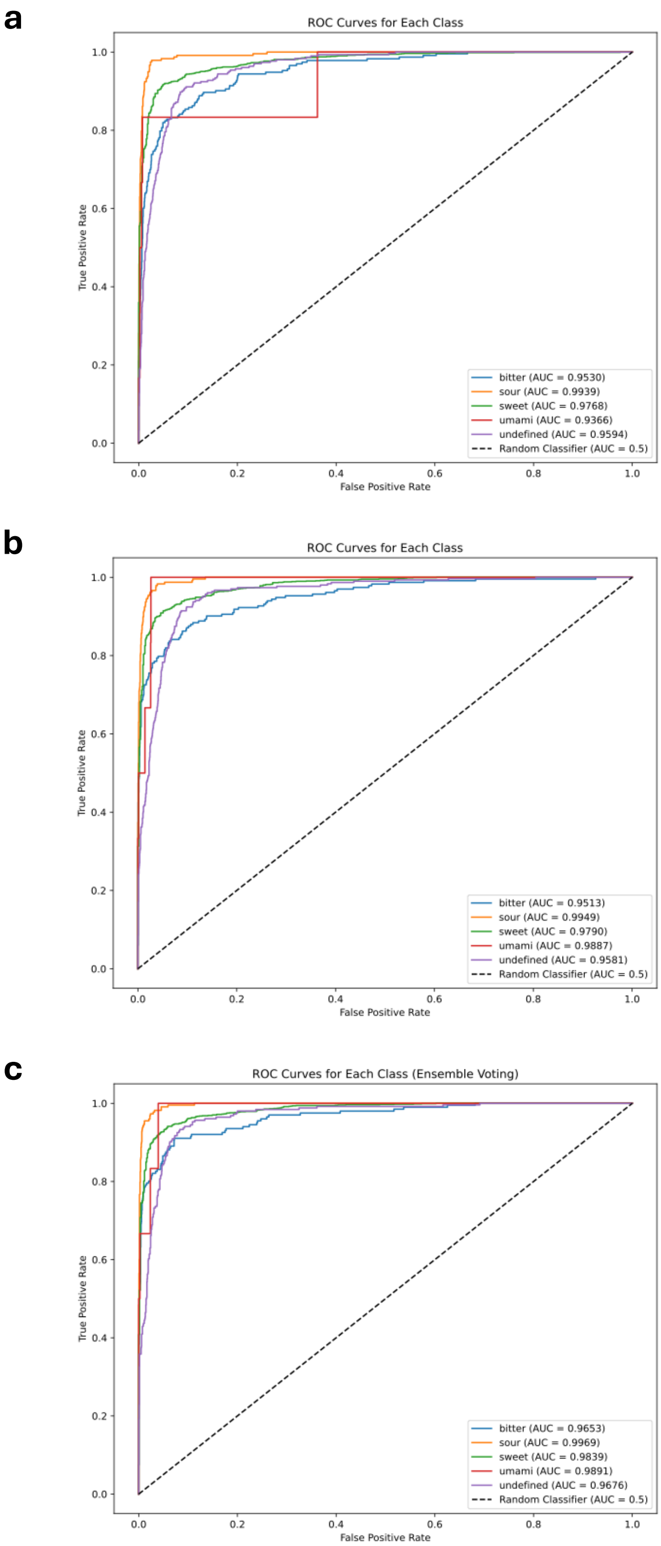

Supplementary Figure 2: Receiver operating characteristics (ROC) for all FART models. (**a**) Using unaugmented training data. (**b**) Using augmented training data. (**c**) Using FART with a confidence metric where predictions on 10 augmented SMILES must agree.

Supplementary Tables 10–11: Unaugmented FART Model Predicting on Non-Canonical SMILES

Supplementary Table 10: Performance of the unaugmented FART model on an augmented test set including non-canonical SMILES. The performance drops markedly compared to an evaluation on only canonical SMILES suggesting that the unaugmented FART has not robustly learned a mapping from structure to taste.

Taste Class	Accuracy	Precision	Recall	F1 Score	AUROC	Support
Bitter		0.5785	0.6289	0.6027		3204
Sour		0.6955	0.8431	0.7622		3224
Sweet		0.9364	0.8759	0.9051		20487
Umami		0.4545	0.0602	0.1064		83
Undefined		0.6583	0.7316	0.6930		3893
Weighted Average		0.8378	0.8265	0.8299	0.9441	
Unweighted Average		0.6646	0.6279	0.6139	0.9317	
Overall Accuracy	0.8265					30891

Supplementary Table 11: Performance of the augmented FART model on an augmented test set including non-canonical SMILES. The performance remains essentially unchanged compared to evaluating on canonical SMILES as predictions are now robust towards non-canonical input.

Taste Class	Accuracy	Precision	Recall	F1 Score	AUROC	Support
Bitter		0.8466	0.7076	0.7709		3198
Sour		0.9007	0.9010	0.9008		3211
Sweet		0.9427	0.9425	0.9426		20520
Umami		0.8889	0.2857	0.4324		84
Undefined		0.6724	0.7741	0.7197		3877
Weighted Average		0.8943	0.8909	0.8911	0.9747	
Unweighted Average		0.8503	0.7222	0.7533	0.9726	
Overall	0.8909					30890

Supplementary Figure 3: Receiver Operating Characteristics for Evaluation on Non-Canonical SMILES

a

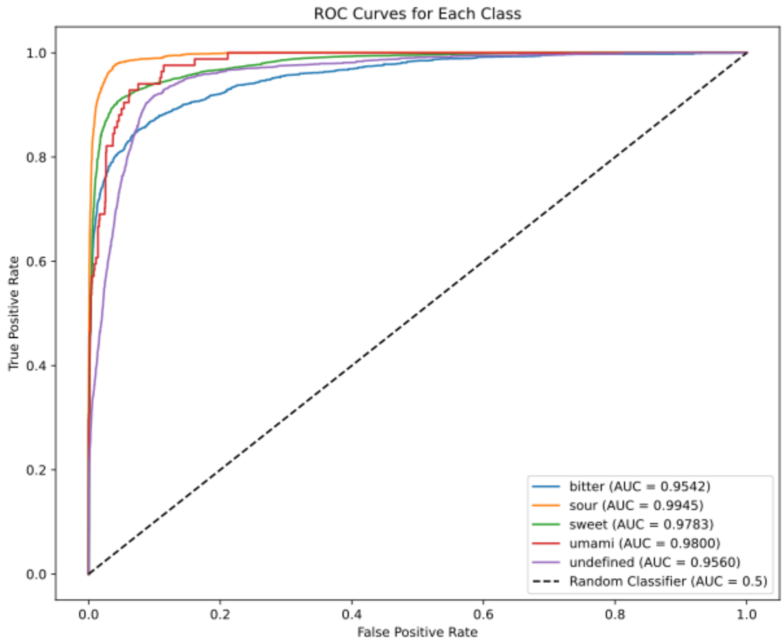

b

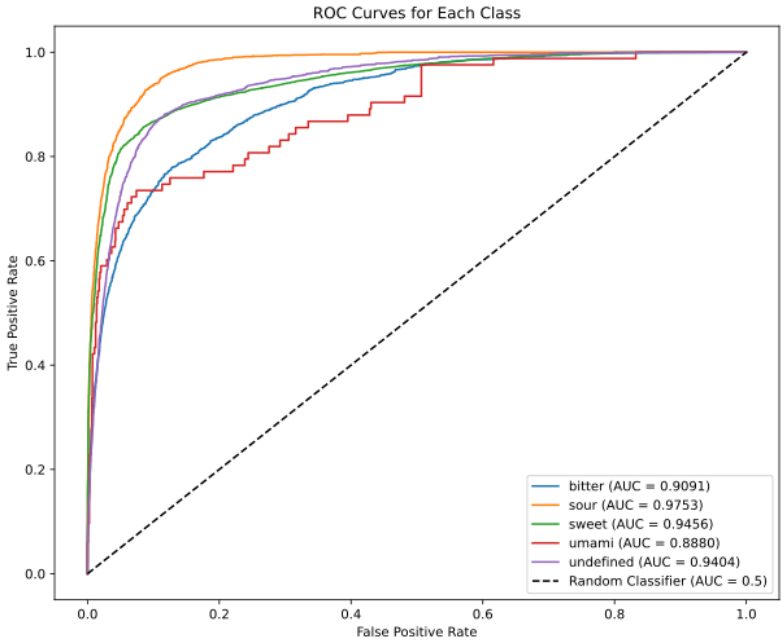

Supplementary Figure 3: (**a**) The FART model trained on augmented SMILES performs well on a test set comprised of both canonical and non-canonical SMILES. (**b**) Performance drops markedly when the model only sees canonical SMILES during training and is then evaluated on non-canonical (i.e. the augmented) SMILES.

Supplementary Figure 4: Analog Generation Based on Explainability Framework

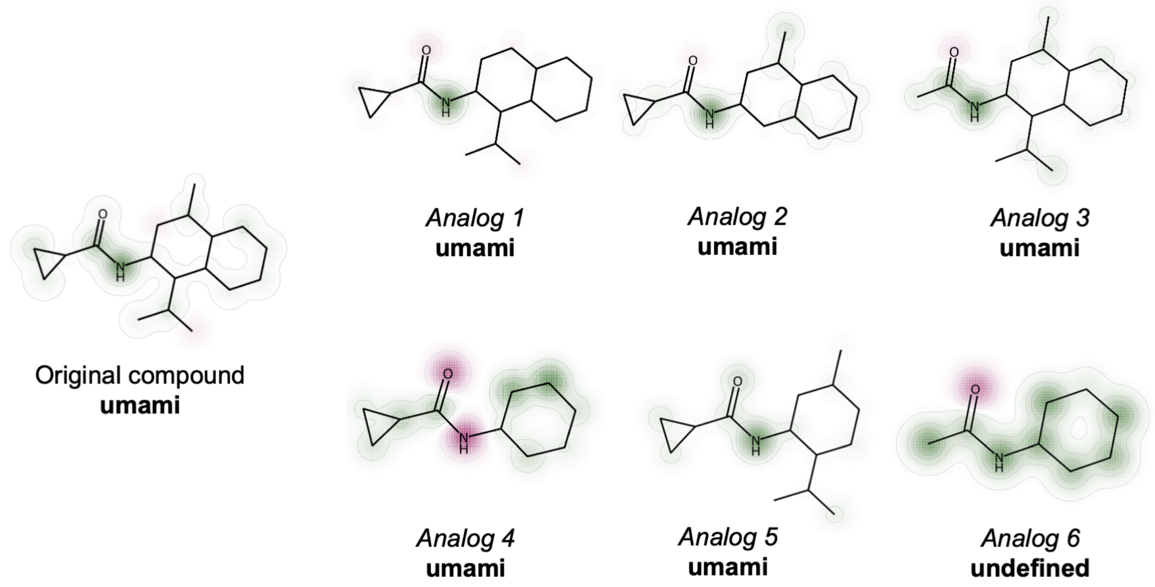

Supplementary Figure 4: To test whether insights from the interpretability framework could be used to generate analogs, we curated six analogs to a previously evaluated molecule. By changing atoms that are less relevant for the umami prediction, most of the analogs retain the umami label.

Supplementary Figure 5: Confusion Matrix Multi-Taste Compounds

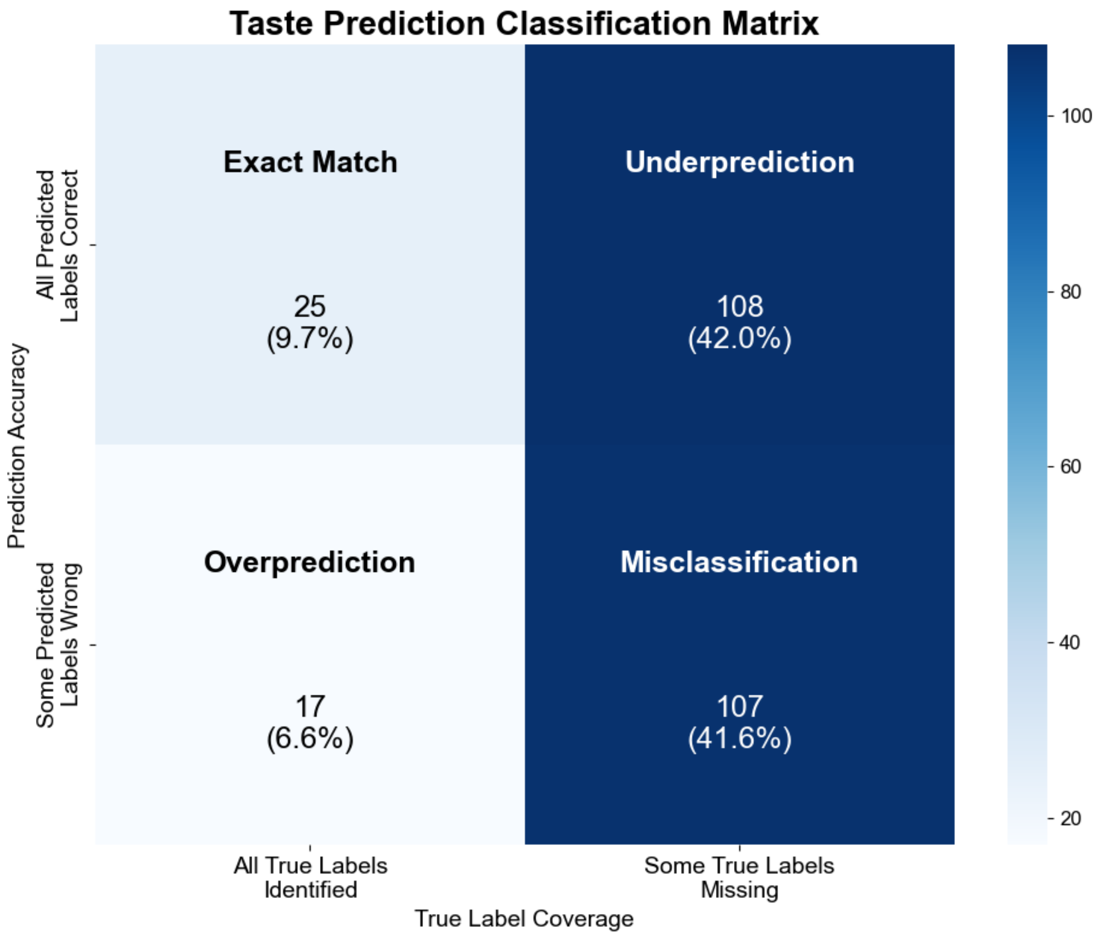

Supplementary Figure 5: A total of 257 molecules had more than one taste label in our training data (excluding undefined). As the FART models do not output multi-labels, instead any logit above 0.2 was considered as a label for this analysis. In many cases, FART still collapses predictions into a single label, which we refer to as underpredicting, i.e. the predicted labels are correct but some true labels are missing. A much rarer case is when the model predicts all correct labels in addition to incorrect labels, i.e. overprediction. A total of 25 molecules are exact matches, the other 107 molecules fall into the misclassification category.
